# Effects of disturbances by forest elephants on diversity of trees and insects in tropical rainforests on Mount Cameroon

**DOI:** 10.1038/s41598-020-78659-7

**Published:** 2020-12-10

**Authors:** Vincent Maicher, Sylvain Delabye, Mercy Murkwe, Jiří Doležal, Jan Altman, Ishmeal N. Kobe, Julie Desmist, Eric B. Fokam, Tomasz Pyrcz, Robert Tropek

**Affiliations:** 1 Institute of Entomology, Biology Centre, Czech Academy of Sciences, Branisovska 31, 37005 Ceske Budejovice, Czech Republic; 2 Faculty of Science, University of South Bohemia, Branisovska 1760, 37005 Ceske Budejovice, Czech Republic; 3 Nicholas School of the Environment, Duke University, 9 Circuit Dr., Durham, NC 27710 USA; 4 Department of Zoology and Animal Physiology, Faculty of Science, University of Buea, P.O. Box 63, Buea, Cameroon; 5 Department of Ecology, Faculty of Science, Charles University, Vinicna 7, 12844 Prague, Czech Republic; 6 Institute of Botany, Czech Academy of Sciences, Dukelska 135, 37982 Trebon, Czech Republic; 7 University Paris-Saclay, 15 rue Georges Clemenceau, 91400 Orsay, France; 8 Institute of Zoology and Biomedical Research, Jagiellonian University, Gronostajowa 9, 30387 Krakow, Poland; 9 Nature Education Centre of the Jagiellonian University, Gronostajowa 5, 30387 Krakow, Poland

**Keywords:** Ecology, Biodiversity, Community ecology, Conservation biology, Forest ecology, Tropical ecology

## Abstract

Natural disturbances are essential for tropical forests biodiversity. In the Afrotropics, megaherbivores have played a key role before their recent decline. Contrastingly to savanna elephants, forest elephants’ impact on ecosystems remains poorly studied. Few decades ago, forests on Mount Cameroon were divided by lava flows, not being crossed by a local population of forest elephants until now. We assessed communities of trees, butterflies and two guilds of moths in the disturbed and undisturbed forests split by the longest lava flow. We surveyed 32 plots, recording 2025 trees of 97 species, and 7853 insects of 437 species. The disturbed forests differed in reduced tree density, height, and high canopy cover, and in increased DBH. Forest elephants’ selective browsing and foraging also decreased tree species richness and altered their composition. The elephant disturbance increased butterfly species richness and had various effects on species richness and composition of the insect groups. These changes were likely caused by disturbance-driven alterations of habitats and species composition of trees. Moreover, the abandonment of forests by elephants led to local declines of range-restricted butterflies. The recent declines of forest elephants across the Afrotropics probably caused similar changes in forest biodiversity and should be reflected by conservation actions.

## Introduction

Natural disturbances are key drivers of biodiversity in many terrestrial ecosystems^1^, including tropical forests despite their traditional view as highly stable ecosystems^2,3^. Natural disturbances such as tree falls, fires, landslides, and insect herbivores outbreaks, generally open forest canopy, followed by temporary changes of microclimate and availability of plant resources (e.g., light, water, and soil nutrients)^4^. The consequent changes in plant communities cause cascade effects on higher trophic levels (herbivores, predators, parasites), expanding the impact of disturbances on the entire ecosystem. Such increase of heterogeneity of habitats and species communities substantially contribute to maintaining the overall biodiversity of tropical forest ecosystems^5,6^.

Megaherbivores, i.e. ≥ 1000 kg herbivorous mammals, used to be among the main causes of such disturbances, before their abundances and diversity seriously dropped in all continents except Africa^7–9^. Among all megaherbivores, savanna elephants are best known to alter their habitats^8,10^. Besides their important roles of seed dispersers or nutrient cyclers^8^, they directly impact savanna ecosystems through disturbing vegetation, especially by increasing tree mortality by browsing, trampling, and debarking^10^. Such habitat alterations substantially affect diversity of many organism groups^11^, including insects. Savanna elephants were shown to positively influence diversity of grasshoppers^12^ and dragonflies^13^, whilst to have ambiguous effect on diversity of particular butterfly families^14,15^. Contrarily, too intensive disturbances caused by savanna elephants impact biodiversity negatively^13,16,17^, similarly to other disturbance types.

Although expected already for a few decades^18^, effects of forest elephants on biodiversity of Afrotropical forests remain strongly understudied^10,19^. Despite being smaller (up to 5 tons, in comparison to 7 tons of savanna elephants), forest elephants are expected to affect their habitats by similar mechanisms as their savanna relatives, as recently reviewed by Poulsen et al*.*^19^. They were shown to impact forest tree density and diversity in both negative and positive ways^19–21^. Besides local opening of forest canopy, they inhibit forest regeneration and maintain small-scaled canopy gaps^22,23^. However, the consequent cascade effects on forest biodiversity have not been studied yet, although effects of elephant disturbances on other tropical forest organisms can be expected as well^24,25^. The only existing study of effects of megafauna, including forest elephants, on the Afrotropical forest invertebrates showed that in defaunated forests, termite abundances decreased by orders of magnitude, followed by a significant decrease of invertebrate contribution to litter decomposition^26^. Such research is urgent especially because of the current steep decline of forest elephants across the Afrotropics (> 60% decrease of abundance between 2002 and 2012^27^), and forest elephants are already extinct in numerous areas, including the protected ones. In such situation, local policy makers and conservationists should be aware of any potential changes in plant and animal communities to initiate more effective conservation planning.

In this study, we bring a direct comparison of a forest structure, and diversity and composition of tree and insect communities in Afrotropical forests with and without forest elephants. Mount Cameroon provides an ideal opportunity for such study by offering a unique ‘natural enclosure experiment’. Forests on its southern slope were split by a continuous lava flow after eruptions in 1982 (from ca 2600 m asl., i.e. above the natural timberline, to ca 1400 m asl.) and 1999 (from ca 1550 m asl. to the seashore)^28^. Probably because of the slow natural succession on this lava flow, local forest elephants do not cross this barrier and stay on its western side close to three crater lakes, the only water sources during the dry seasons^28^. Such unusual conditions represent a long-term (at least since the last eruption in 1999 in the lower elevations, and since the eruption in 1982 in the upper elevations) enclosure experiment under natural conditions, performed on a much larger scale than any possible artificial enclosure studies. In the disturbed and undisturbed sites, we surveyed forest structure and communities of trees, butterflies, and two ecological guilds of moths. We hypothesized that forest elephants changed the forest structure by opening its canopy, with the consequent changes in composition of all studied groups’ communities. We expected decrease of tree diversity by the direct damage by elephants, and related increase of insect diversity caused by the higher habitat heterogeneity. Nevertheless, butterflies and moths differ in their habitat use and requirements. Because butterflies mostly rely on a direct solar radiation for their thermoregulation and other activities, their diversity have been shown to be mostly influenced by forest structure and canopy openness in tropical forests^29^. On the other hand, moths are relatively less dependent on their habitat structure due to the nocturnal behaviour, and they are more affected by the plant community composition^29,30^. Therefore, the ambiguous effect can be also hypothesized, as moths more closely depend on tree diversity^31^, whilst butterflies rather benefit from canopy opening^32^. Finally, we focus on species’ distribution ranges in both types of forests, with no a priori hypothesis on the direction of the changes.

## Results

In total, 2025 trees were identified to 97 species and 7853 butterflies and moths were identified to 437 species in all sampled forest plots (Supplementary Table S1).

### Elephant disturbances and forest structure

**Figure 1:**
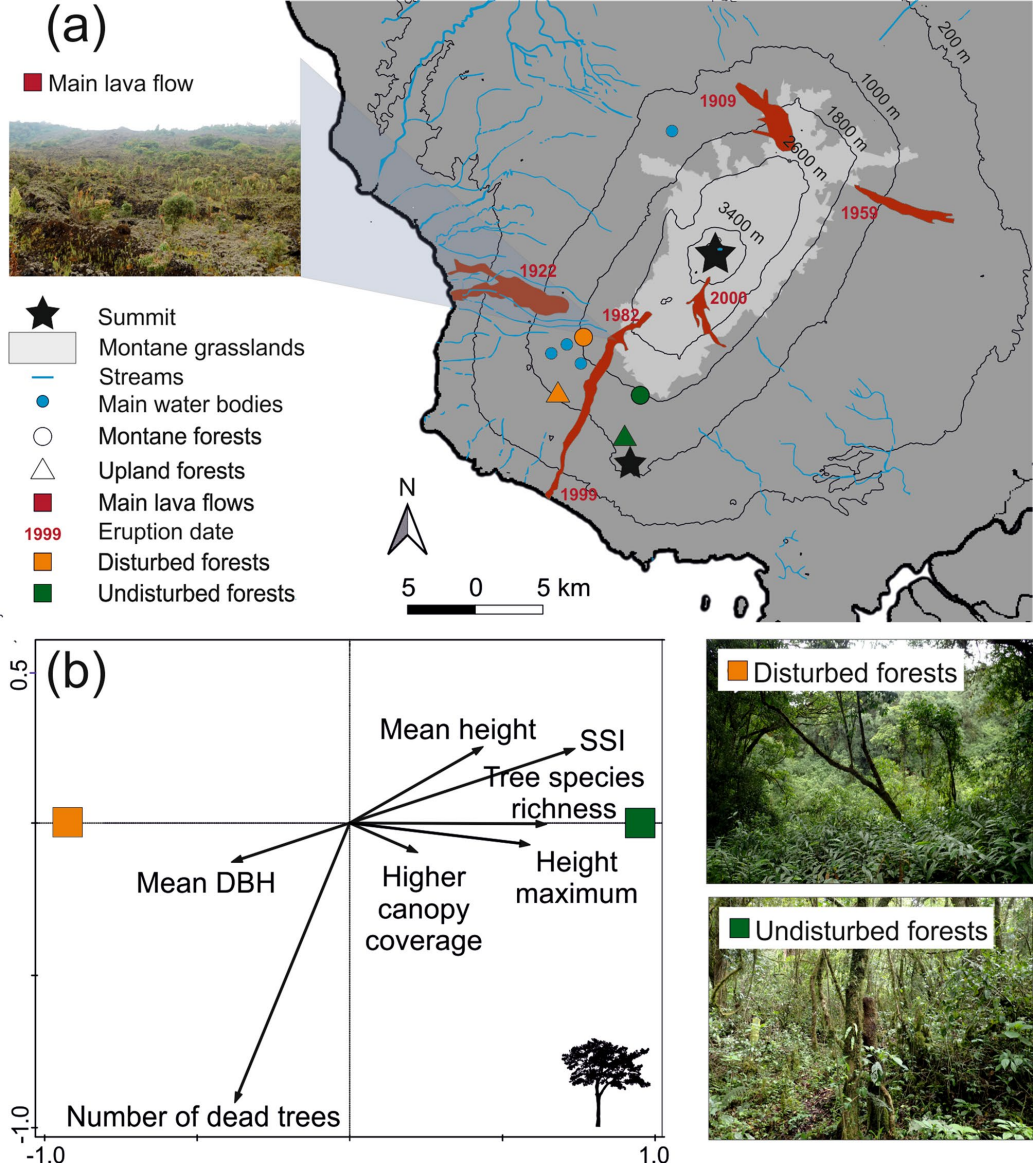
(**a**) Map of Mount Cameroon with the main lava flows, sampled forests and water resources. The background map was created in QGIS v. 3.10.0 ‘A Coruña’ (https://qgis.org) and Corel Draw X7 (https://www.coreldraw.com). The pictures of disturbed and undisturbed forests were taken at the studied montane sites. (**b**) Redundancy analysis diagram visualizing effects of disturbances by elephants on forest structure.

The partial-RDA ordination analysis showed significant differences in the forest structure descriptors between the disturbed and undisturbed forests (Fig. 1b). In total, the two main ordination axes explained 18.5% of the adjusted variation (all axes eigenvalues: 0.83; Pseudo-F = 7.8; p = 0.002). In the disturbed plots, *tree species richness*, *mean SSI*, *mean height*, *maximum height*, and *higher canopy coverage* were lower. In contrast, *mean DBH* was larger in the disturbed forests (Fig. 1b).

### Elephant disturbances and tree diversity

**Figure 2:**
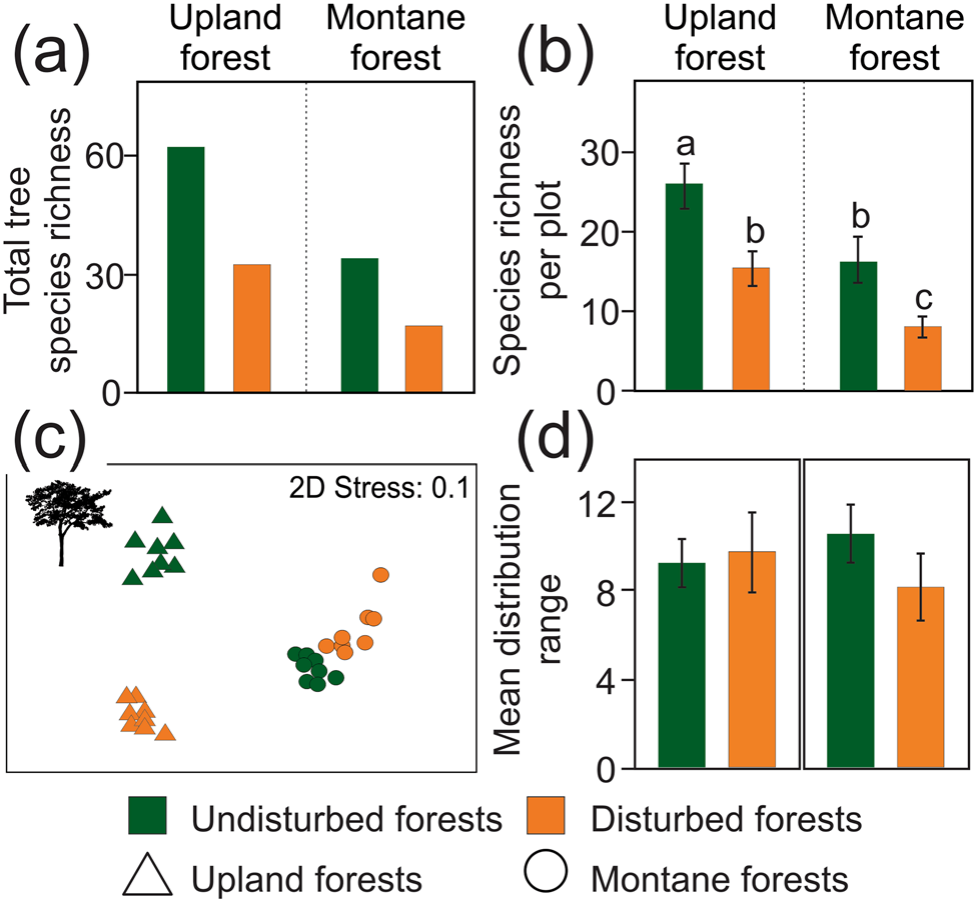
Differences in tree species richness, community composition, and mean distribution range between forests disturbed and undisturbed by elephants. Tree species richness per (**a**) forest site, and (**b**) per sampling plot estimated by GEE (estimated means with 95% unconditional confidence intervals). The letters visualize results of the post-hoc pairwise comparisons. (**c**) NMDS diagrams of the tree community compositions at the sampled forest plots. (**d**) Mean distribution range of trees per sampling plot estimated by GEE.

Elephant disturbances affected tree species richness per sampled elevation, as well as per sampled plot. In both upland and montane forests, total tree species richness of the disturbed sites was nearly half in comparison to the undisturbed sites (Fig. 2a; Supplementary Table S1). Tree species richness per plot was significantly affected by disturbance (higher at undisturbed forest plots) and elevation (higher at the upland forests) (Fig. 2b; Table 1). The responses of individual tree families were significantly affected by disturbance and elevation (Pseudo-F: 12.3, p-value: < 0.001, adjusted explained variation: 52.3%; Supplementary Table S2). All tree families but Euphorbiaceae showed higher species richness at undisturbed forests; all families but Rubiaceae had higher species richness in upland forests (Supplementary Fig. S1a).

**Table 1:** Results of the GEE models analyzing effects of disturbance, season and elevation on species richness and mean distribution range of trees and insects in forests disturbed and undisturbed by elephants on Mount Cameroon.

Focal group	Tested variable	Species richness			Distribution range		
		df	Wald χ^2^	p-value^a^	df	Wald χ^2^	p-value^a^
Trees	Disturbance	1	21.9	< 0.001***	1	1.4	0.23
	Elevation	1	51.9	< 0.001***	1	0	0.86
	Disturbance × Elevation	1	1.3	0.25	1	3.9	0.05*
Butterflies	Disturbance	1	4.7	0.031*	1	9.5	0.002**
	Season	1	0	0.964	1	67.6	< 0.001***
	Elevation	1	10.2	0.001**	1	2.5	0.115
	Disturbance × Season	1	7.4	0.007**	1	0.2	0.654
	Disturbance × Elevation	1	45.1	< 0.001***	1	7.3	0.007**
Fruit-feeding moths	Disturbance	1	3.3	0.069	–	–	–
	Season	1	3.2	0.072	–	–	–
	Elevation	1	27.3	< 0.001***	–	–	–
	Disturbance × Season	1	149.7	< 0.001***	–	–	–
	Disturbance × Elevation	1	7.2	0.007**	–	–	–
Light-attracted moths	Disturbance	1	6.2	0.012*	1	5.1	0.024*
	Season	1	2.5	0.112	1	0.8	0.372
	Elevation	1	2.4	0.123	1	6.9	0.009**
	Disturbance × Season	1	8.9	0.003**	1	0.5	0.462
	Disturbance × Elevation	1	67.0	< 0.001***	1	12.4	< 0.001**

^a^ *p < 0.05; **p < 0.01; ***p < 0.001.

Tree communities significantly differed in composition between the forests disturbed and undisturbed by elephants according to the partial-CCA (all-axes eigenvalues: 4.55; Pseudo-F = 3.8; p < 0.001). The first NMDS axis reflected elevation, whilst the tree communities of the disturbed and undisturbed forests were relatively well-separated along the second axis (Fig. 2c). The ordination diagram also showed relatively higher dissimilarities of tree communities between the disturbed and undisturbed plots at the upland than at the montane forests (Fig. 2c).

### Elephant disturbances and insect diversity

**Figure 3:**
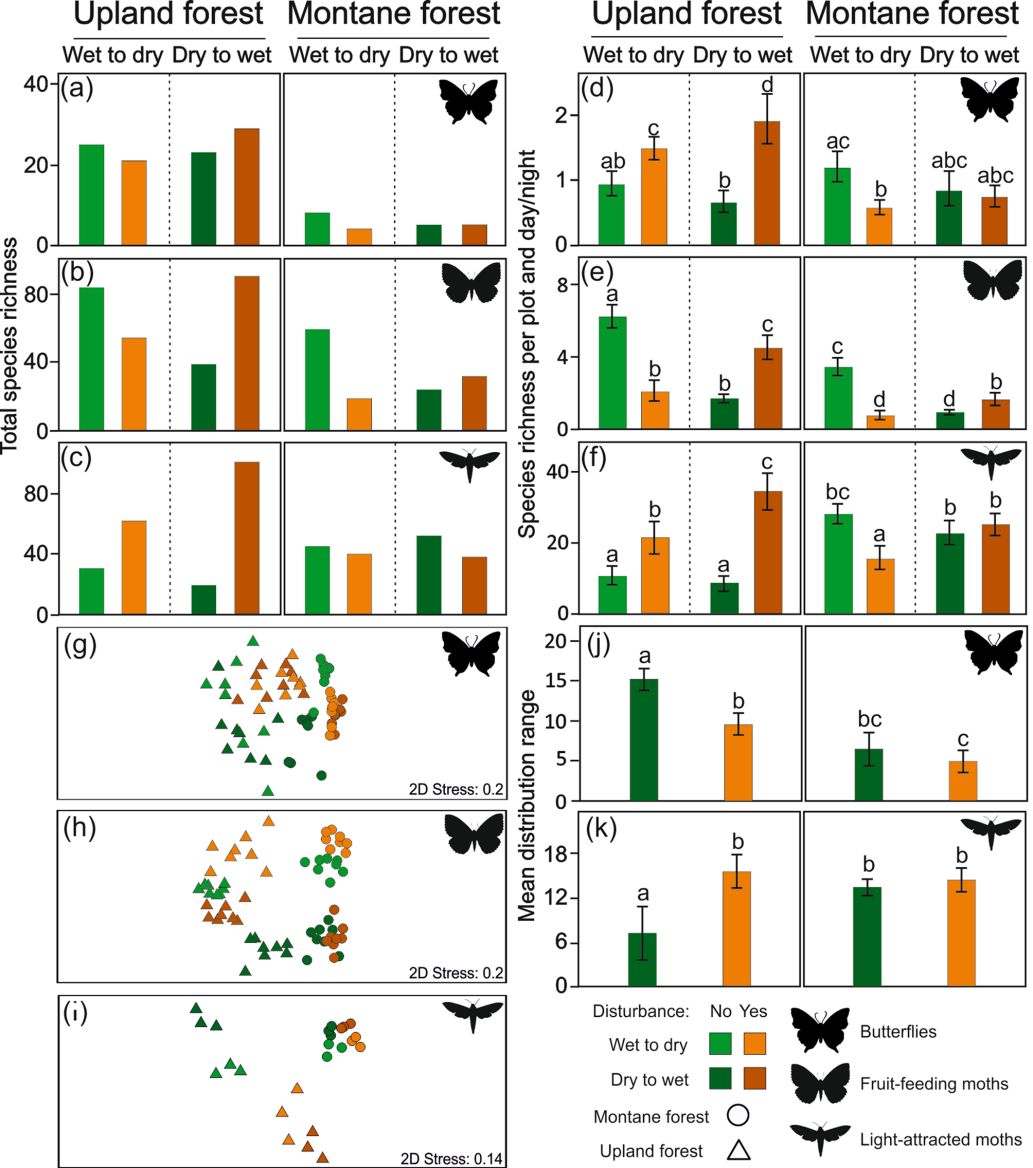
Species richness of insects per sampling site and season (**a**–**c**), and sampling plots and day or night (**d**–**f**) as estimated by GEEs (estimated means with 95% unconditional confidence intervals are visualized). (**g**–**i**) NMDS diagrams of insect community compositions at the sampled forest plots. (**j**,**k**) Mean distribution range of insects estimated by GEEs. Letters visualize results of the post-hoc pairwise comparisons.

The responses of individual insect groups’ total species richness per sampling site to elephant disturbances were rather inconsistent among the studied elevations and seasons. Butterflies and fruit-feeding moths showed lower total species richness in the disturbed forests at both elevations during the transition from wet to dry seasons, which became higher or comparable to the undisturbed forests during the transition from dry to wet seasons (Fig. 3a,b; Supplementary Table S1). Light-attracted moths were species-richer in the disturbed upland forest than in the undisturbed upland forest during both sampled seasons but species-poorer in the montane forest during both sampled seasons (Fig. 3c; Supplementary Table S1).

The effects of elephant disturbances on insect species richness per plot also differed among the studied insect groups. The interactions disturbance × season and disturbance × elevation were significant for all insect groups (Table 1), indicating complex effects of elephant disturbances on insect species richness. GEEs showed a significant positive effect of elephant disturbances on species richness of butterflies and light-attracted moths (Fig. 3d,f; Table 1). No significant effect of elephant disturbances was detected for fruit-feeding moths (Table 1). Both butterflies and fruit-feeding moths were significantly species richer at the lower altitudes, whilst no significant effect of elevation on light-attracted moths was revealed (Fig. 3d–f; Table 1). Insignificant effects of season were shown for all studied insect groups (Table 1). For butterflies and light-attracted moths, the pairwise post-hoc comparisons of disturbed and undisturbed forests showed that species richness was significantly higher in the disturbed upland forests for both groups, and significantly lower or not significantly different (depending on the sampled season) in the montane forests (Fig. 3d,f). In contrast, fruit-feeding moth species richness was significantly lower in the disturbed forests at both elevations during the transition from wet to dry season, but significantly richer during the transition from dry to wet season (Fig. 3e). The analyses of individual family species richness showed significant effects of the disturbance × season interaction for butterflies and light-attracted moths (butterflies: Pseudo-F: 12.8, p-value: < 0.001, adjusted explained variation: 36.4%; light-attracted moths: Pseudo-F: 15.8, p-value: < 0.001, adjusted explained variation: 49.1%; Supplementary Table S2). Whilst for butterflies, all families showed consistently higher species richness at disturbed upland forests, most of the analysed families of light-attracted moths had the highest species richness at undisturbed montane forest (Supplementary Fig. S1b,c). The only exceptions were Notodontidae with higher species richness at disturbed upland forest, and Lymantriinae with no apparent trend (Supplementary Fig. S1c).

Elephant disturbances significantly affected species composition of all focal insect groups in partial CCAs (butterflies: all-axes eigenvalue: 2.75; Pseudo-F: 4.6; p-value: < 0.001; fruit-feeding moths: all-axes eigenvalue: 5.27; Pseudo-F: 3.2; p-value: < 0.001; light-attracted moths: all-axes eigenvalue: 2.96; Pseudo-F: 4.5; p-value: < 0.001). For butterflies and fruit-feeding moths, the first NMDS axes can be related to elevation, in contrast to light-attracted moths where elevation can be related to the second NMDS axis (Fig. 3g–i). All groups were well-clustered according to the disturbance type at both elevations. The effect of disturbance was interacting with season and elevation for all groups (Fig. 3g–i). Among all insect groups, light-attracted moths species composition responded to elephant disturbances very similarly to trees, with well-separated upland disturbed and undisturbed forest types and comparatively less heterogenous montane forest samples (Figs. 2c, 3i).

### Elephant disturbances and species’ distribution range

Elephant disturbances and elevation showed marginally significant effects of their interaction on distribution range of tree species, although no significant separate effect was detected for them (Table 1). In the undisturbed forests, the mean tree species’ distribution range was positively associated with increasing elevation, while negatively associated with increasing elevation in the disturbed forests. However, the pairwise post-hoc comparisons were insignificant (Fig. 2d).

Patterns of distribution range differed between the two analysed insect groups. Butterfly species’ distribution range was significantly lower at high elevation and in the disturbed forests (Fig. 3j). Similarly, moths’ mean distribution range was significantly affected by elephant disturbances and seasons (Table 1). Nevertheless, pairwise post-hoc comparisons showed that light-attracted moths in the undisturbed upland forest had a significantly lower distribution range than in all other studied forests, which did not significantly differ from each other (Fig. 3k).

## Discussion

Our study has shown a strong effect of forest elephants on tropical forest biodiversity. Concordant to our first hypothesis, their long-term absence at the studied forests changed the forest structure. It has led to an increase of forest height, closure of its canopy, and dominance of smaller over large trees. This observed shift in forest structure can be interpreted by a combination of direct and indirect effects driven by forest elephants. Because of their high appetite and large body size, forest elephants surely eliminate some trees^23^. They directly consume high amount of tree biomass, as well as their fruits and seeds^33,34^. When struggling through forest, elephants break stems and sometimes even uproot trees, while their repeated trampling denude the forest floor and destroy fallen seeds and saplings^23^. Moreover, the direct damages are likely to increase tree susceptibility to pathogens or decomposers, as shown in the previous study of termites^26^. Although the number of dead trees seemed to poorly characterize the disturbed forests (potentially because of the significantly higher decomposition in elephant-disturbed forests^26^), the higher tree density in the undisturbed plots supports this hypothesis. Thus, the presence of a few large trees in the plots disturbed by forest elephants can be explained by only a small portion of trees escaping the browsing pressure^23^.

Together with altering the forest structure, forest elephants decreased tree species richness and change tree community composition, confirming our second hypothesis. Although forest elephants are generalized herbivores, they prefer particular tree and other plant species^34^. Thereby, their selective browsing of palatable species affects tree mortality and recruitment, which can explain the observed differences in tree communities between the disturbed and undisturbed forests. Finally, similarly as in savanna, we can reasonably expect different resistance of tree species to repeated disturbances by forest elephants, or differences in their ability to recover from damages^35^. The revealed higher species richness of Euphorbiaceae trees to the disturbed forest could indicate some resistance to elephant disturbances. Unfortunately, the knowledge of African forest elephants’ browsing preferences and/or Afrotropical trees’ resistance to disturbances are not enough to decide which effect prevails in the alterations of forest structure by elephants.

The presence of forest elephants impacted all studied herbivorous insect communities as well, although differently for particular insect groups. These can be related to the changes in composition of tree communities and in habitat structure in the disturbed forests. The upland forests disturbed by elephants harboured more species of butterflies and light-attracted moths. This effect was consistent for all butterfly families, although only for notodontid moths. However, all other effects of disturbances differed according the studied elevation and season, as well as among the insect groups. Many tropical butterflies rely on forests gaps and solar radiation for their thermoregulation^36^ and oviposition on larval food-plants (mostly herbs^37^), therefore their diversity decrease after the upland forest elephant’s enclosure cannot be surprising. By opening of forest canopy, forest elephants could support quantity and heterogeneity of resources available for butterflies. However, such hypothesis can hardly explain the detected decrease of light-attracted (night flying) moth diversity in the undisturbed upland forests. In fact, diversity of moths has been repeatedly shown to increase with diversity of trees, as the most common food plants for their caterpillars^38,39^. Therefore, the opposite effect of disturbance by forest elephants can be expected, this was confirmed at least for most light-attracted moth families in the montane forests. Unfortunately, we do not have any other explanation of the positive effect of forest disturbances in the sampled upland forests. Contrastingly, fruit-feeding moths are relatively independent to forest structure^29^. They can follow the spatiotemporal changes of ripe fruits (adult food) or young sprouts (larval food) more tightly than fruit-feeding butterflies, which could partly explain their seasonally inconsistent reaction to the elephant disturbances. Unfortunately, no data to confirm or reject such hypothesis exist.

In the montane forests, we found no consistent changes of the insects’ diversity, as it strongly varied with season and studied insect group. Moreover, the communities of all insect groups were highly homogeneous in both forest types in this high elevation. The montane forests on Mount Cameroon are already relatively open and with limited tree diversity^40^ that additional disturbances by elephants could hardly increase habitat heterogeneity even for butterflies. Moreover, some tree dominants in the montane forests, such as *Schefflera abyssinica* and *S. mannii*, are (semi)deciduous during the dry season which generally open the higher canopy even in the undisturbed forests. Simultaneously, these dominants get typically recruited as epiphytes, later strangling their hosts^41^. Therefore, they may more efficiently escape from any elephant effects. We hypothesize that these effects together result in more similarity between the disturbed and undisturbed forests at higher elevations. Last but not least, we have recently revealed a strong seasonal shift in elevational ranges of both butterflies and moths^42^; the seasonal discrepancies in the effect of disturbance could be related to it. Unfortunately, we do not have any detailed data on this phenomenon from the undisturbed forest plots.

Recently, Poulsen et al*.*^19^ discussed the fate of Afrotropical forests in the future world without forest elephants. The authors hypothesized that their loss would increase understory stem density and change tree species composition. We concur with Poulsen’s hypotheses from our data study. Moreover, we have shown that the change of forest structure and composition can have strong cascading effects on other trophic levels, at least in the upland forests. Hawthorne and Parren^21^ demonstrated that the disappearance of forest elephants from several Ghanaian forests did not have any remarkable effect on plant populations at the country level. However, our study has shown that the local consequences of forest elephants’ disappearance can be highly significant for trees, as well as for higher trophic levels.

Natural disturbances are important ecological processes increasing habitat heterogeneity^6,43^. Interestingly, our results showed that some groups of trees and insects may respond positively to the natural disturbances by forest elephants, whilst some others respond negatively. Because many tropical species have highly specific habitat needs^6,29,44^, homogeneous non-disturbed ecosystems could be impoverished for such open-habitat specialists, whilst the too disturbed ecosystems would lack the close-canopy forest species. Therefore, only the dynamically disturbed ecosystems could harbour the ‘complete’ local biodiversity. Although more comparative studies are required, forest elephant extinction would accelerate the vegetation succession, enclose the forest canopy, and generally impoverish the habitat heterogeneity in Afrotropical forests. These would be unavoidably followed by changes in tropical forest communities and by declines of range-restricted species that profit from disturbances, as we have shown for some of the herbivorous insects in the upland forests.

In conclusion, our study showed that African forest elephants contribute for maintaining the tropical forest heterogeneity and tree diversity. The elephant-related habitat heterogeneity increased the heterogeneity of available niches and sustain diverse communities of Afrotropical insects. Despite the lack of any data, we can speculate on consequences for biodiversity at other trophic levels. Nevertheless, we have confirmed the African forest elephant as a key-stone species in the Afrotropical forest ecosystems. Altogether, the maintenance of forest elephant populations in Afrotropical forests appears to be necessary to prevent biodiversity declines. Unfortunately, the decline of forest elephant populations in West and Central African tropical forests is alarming, and most probably have already been followed by other species extinctions. It is even highly probable that such processes are already ongoing, although unrecorded in one of the least studied biogeographic areas in the world. Therefore, we urge for more efficient conservation of the remaining populations of forest elephants. Their effects on the entire tropical forest ecosystems must be recognized and incorporated into the management plans of Afrotropical protected areas.

## Methods

### Study area

Mount Cameroon (South-Western Province, Cameroon) is the highest mountain in West/Central Africa. This active volcano rises from the Gulf of Guinea seashore up to 4095 m asl. Its southwestern slope represents the only complete altitudinal gradient of primary forests from lowland up to the timberline (~ 2200 m asl.) in the Afrotropics. Belonging to the biodiversity hotspot, Mount Cameroon harbour numerous endemics^45–47^. With > 12,000 mm of yearly precipitation, foothills of Mount Cameroon belong among the globally wettest places^42^. Most precipitation occur during the wet season (June–September; > 2000 mm monthly), whilst the dry season (late December–February) usually lacks any strong rains^42^. Since 2009, most of its forests have become protected by the Mount Cameroon National Park.

Volcanism is the strongest natural disturbance on Mount Cameroon with the frequency of eruptions every ten to thirty years. Remarkably, on the studied southwestern slope, two eruptions in 1982 and 1999 created a continuous strip of bare lava rocks (in this study referred as ‘the lava flow’) interrupting the forests on the southwestern slope from above the timberline down to the seashore (Fig. 1a).

A small population of forest elephants (*Loxodonta cyclotis*) strongly affects forests above ca. 800 m asl. on the southwestern slope^28,45^. It is highly isolated from the nearest populations of the Korup NP and the Banyang-Mbo Wildlife Sanctuary, as well as from much larger metapopulations in the Congo Basin^48^. It has been estimated to ~ 130 individuals with a patchy local distribution^28^. On the southwestern slope, they concentrate around three crater lakes representing the only available water sources during the high dry season, although their local elevational range covers the gradient from lowlands to montane grasslands just above the timberline^28^. They rarely (if ever) cross the old lava flows, representing natural obstacles dividing forests of the southwestern slope to two blocks with different dynamics. As a result, forests on the western side of the longest lava flow have an open structure, with numerous extensive clearings and ‘elephant pastures’, whereas eastern forests are characteristic by undisturbed dense canopy (Fig. 1). To our knowledge, the two forest blocks are not influenced by any extensive human activities, nor differ in any significant environmental conditions^28,45^. Hereafter, we refer the forests west and east from the lava flow as *disturbed* and *undisturbed*, respectively. Effects of forest elephant disturbances on communities of trees and insects were investigated at four localities, two in an upland forest (1100 m asl.), and two in a montane forest (1850 m asl.).

### Tree diversity and forest structure

At each of four sampling sites, eight circular plots (20 m radius, ~ 150 m from each other) were established in high canopy forests (although sparse in the undisturbed sites), any larger clearings were avoided. In the disturbed forest sites, the plots were previously used for a study of elevational diversity patterns^40,42^. In the undisturbed forest sites, plots were established specifically for this study.

To assess the tree diversity in both disturbed and undisturbed forest plots, all living and dead trees with diameter at breast height (DBH, 1.3 m) ≥ 10 cm were identified to (morpho)species (see^40^ for details). To study impact of elephant disturbances on forest structure, each plot was characterized by twelve descriptors. Besides *tree species richness*, *living* and *dead trees* with DBH ≥ 10 cm were counted. Consequently, DBH and basal area of each tree were measured and averaged per plot (*mean DBH* and *mean basal area*). Height of each tree was estimated and averaged per plot (*mean height*), together with the tallest tree height (*maximum height*) per plot. From these measurements, two additional indices were computed for each tree: stem slenderness index (SSI) was calculated as a ratio between tree height and DBH, and tree volume was estimated from the tree height and basal area^49^. Both measurements were then averaged per plot (*mean SSI* and *mean tree volume*). Finally, following Grote^50^, proxies of shrub, lower canopy, and higher canopy coverages per plot were estimated by summing the DBH of three tree height categories: 0–8 m (shrubs), 8–16 m (lower canopy), > 16 m (higher canopy).

### Insect sampling

Butterflies and moths (Lepidoptera) were selected as the focal insect groups because they belong into one of the species richest insect orders, with relatively well-known ecology and taxonomy, and with well-standardized quantitative sampling methods. Moreover, they strongly differ in their habitat use^29^. In conclusion, butterflies^51^ and moths^52^ are often used as efficient bioindicators of changes in tropical forest ecosystems, especially useful if both groups are combined in a single study. Within each sampling plot, fruit-feeding lepidopterans were sampled by five bait traps (four in understory and one in canopy per sampling, i.e. 40 traps per sampling site, and 160 traps in total) baited by fermented bananas (see Maicher et al*.*^42^ for details). All fruit-feeding butterflies and moths (hereinafter referred as *butterflies* and *fruit-feeding moths*) were killed (this is necessary to avoid repetitive counting of the same individuals^53^) daily for ten consecutive days and identified to (morpho)species.

Additionally, moths were attracted by light at three ‘mothing plots’ per sampling site, established out of the sampling plots described above. These plots were selected to characterize the local heterogeneity of forest habitats and separated by a few hundred meters from each other. To keep the necessary standardisation, all mothing plots at both types of forest were established in semi-open patches, avoiding both dense forest and larger openings. Moths were attracted by a single light (see Maicher et al*.*^42^ for details) during each of six complete nights per elevation (i.e., two nights per plot). Six target moth groups (Lymantriinae, Notodontidae, Lasiocampidae, Sphingidae, Saturniidae, and Eupterotidae; hereafter referred as *light-attracted moths*) were collected manually, killed, and later identified into (morpho)species. The three lepidopteran datasets (butterflies, and fruit-feeding and light-attracted moths) were extracted from Maicher et al*.*^42^ for the disturbed forest plots, whilst the described sampling was performed in the undisturbed forest plots specifically for this study. Voucher specimens were deposited in the Nature Education Centre, Jagiellonian University, Kraków, Poland.

To partially cover the seasonality^54^, the insect sampling was repeated during transition from wet to dry season (November/December), and transition from dry to wet season (April/May) in all disturbed and undisturbed forest plots.

### Diversity analyses

To check sampling completeness of all focal groups, the sampling coverages were computed to evaluate our data quality using the *iNEXT* package^55^ in R 3.5.1^56^. For all focal groups in all seasons and at all elevations, the sampling coverages were always ≥ 0.84 (mostly even ≥ 0.90), indicating a sufficient coverage of the sampled communities (Supplementary Table S1). Therefore, observed species richness was used in all analyses^57^.

Effects of *disturbance* on species richness were analysed separately for each focal group by Generalized Estimated Equations (GEE) using the *geepack* package^58^. For trees, species richness from individual plots were used as a ‘sample’ with an independent covariance structure, with *disturbance*, *elevation*, and their interaction treated as explanatory variables. For lepidopterans, because of the temporal pseudo-replicative sampling design, species richness from a sampling day (butterflies and fruit-feeding moths) or night (light-attracted moths) at individual plot was used as a ‘sample’ with the first-order autoregressive relationship *AR*(*1*) covariance structure (i.e. repeated measurements design). *Disturbance*, *season*, *elevation*, *disturbance* × *season*, and *disturbance* × *elevation* were treated as explanatory variables. All models were conducted with Poisson distribution and log-link function. Pairwise post-hoc comparisons of the estimated marginal means were compared by Wald χ^2^ tests. Additionally, species richness of individual families of trees, butterflies, and light-attracted moths were analysed by Redundancy Analyses (RDA), a multivariate analogue of regression, based on the length of gradients in the data^59^. All families with > 5 species were included in three RDA models, separately for the studied groups (the subfamily name Lymantriinae is used, because they are the only group of the hyperdiverse Erebidae family of the light-attracted moths). Fruit-feeding moth families were not analyzed because 83% of their specimens belonged to Erebidae and all other families were therefore minor in the sampled data. Species richness of individual families per plot were used as response variables, whilst interaction of *disturbance* and *elevation* were applied as factorial explanatory variable (for butterflies and light-attracted moths, the temporal variation was treated by adding *season* as a covariate).

Differences in composition of communities between the disturbed and undisturbed forests were analysed by multivariate ordination methods^59^, separately for each focal group. Firstly, the main patterns in species composition of individual plots were visualized by Non-Metric Multidimensional Scaling (NMDS) in Primer-E v6^60^. NMDSs were generated using Bray–Curtis similarity, computed from square-root transformed species abundances per plot. Subsequently, influence of *disturbance* on community composition of each focal group was tested by constrained partial Canonical Correspondence Analyses (CCA) with log‐transformed species’ abundances as response variables and *elevation* as covariate^59^. Significance of all partial CCAs were tested by Monte Carlo permutation tests with 9999 permutations.

Finally, differences in the forest structure descriptors between the disturbed and undisturbed forests were analysed by partial Redundancy Analysis (RDA). Prior to the analysis, preliminary checking of the multicollinearity table among the structure descriptors was investigated. Only forest structure descriptors with pairwise collinearity < 0.80, i.e. tree species richness, number of dead trees, mean DBH, mean height, maximum height, mean SSI, and higher canopy coverage, were included in these analyses. Their log‐transformed values were used as response variables^59^. RDA was then run with *disturbance* as explanatory variable and *elevation* as covariate, and tested by Monte Carlo permutation test (9999 permutations). All CCAs and RDAs were performed in Canoco 5^61^.

### Species distribution range

To analyse if the elephant disturbance supports rather range-restricted species or widely distributed generalists, we used numbers of Afrotropical countries with known records of each tree and lepidopteran species as a proxy for their distribution range; we are not aware of any more precise existing dataset covering all studied groups for the generally understudied Afrotropics. Because of the limited knowledge on Afrotropical Lepidoptera, we ranked only butterflies and light-attracted Sphingidae and Saturniidae moths (the latter two analysed together and referred as *light-attracted moths*). This distribution data were excerpted from the RAINBIO database for trees^62^, Williams^63^ for butterflies, and Afromoths.net for moths^64^; all considered as the most comprehensive databases. Two non-native trees (*Persea americana* and *Cecropia peltata*) and three tree species not included in the RAINBIO database and all morphospecies were excluded from these analyses. In total, 73 species of trees and 71 species of insects (50 butterflies and 21 moths) were included in the distribution range analyses.

To consider the relative abundances of individual species in the communities, the distribution range of each species was multiplied by the number of collected individuals per sample and their sums were divided by the total number of individuals recorded at each sample. These *mean distribution ranges* per sample were then compared between disturbed and undisturbed forest sites by GEE analyses (with normal distribution; independent covariance structure) following the same model design as for the above-described comparisons of species richness.

## Supplementary Information

Supplementary Information.

## Data Availability

Data available via the Zenodo repository (10.5281/zenodo.4300119).
