# Supplementary material for: Effects of disturbances by forest elephants on diversity of trees and insects in tropical rainforests on Mount Cameroon
Source: Sci Rep. 2020 Dec 10;10:21618. doi: 10.1038/s41598-020-78659-7 (PMC7729851; doi:10.1038/s41598-020-78659-7)
Supplement: Supplementary file 1 — Supplementary Information. [file 41598_2020_78659_MOESM1_ESM.docx]

*Scientific Reports*

**SUPPORTING INFORMATION**

**Effects of disturbances by forest elephants on diversity of trees and insects in tropical rainforests on Mount Cameroon**

Vincent Maicher, Sylvain Delabye, Mercy Murkwe, Jiří Doležal, Jan Altman, Ishmeal N. Kobe, Julie Desmist, Eric B. Fokam, Tomasz Pyrcz, Robert Tropek

**Table S1.** Abundances and diversity of trees and insects in forests disturbed and undisturbed by elephants in particular seasons.

|  |  | **Undisturbed forests** | | | | |  | **Disturbed forest** | | | | |
| --- | --- | --- | --- | --- | --- | --- | --- | --- | --- | --- | --- | --- |
|  |  | **1,100 m a.s.l.** | |  | **1,850 m a.s.l.** | |  | **1,100 m a.s.l.** | |  | **1,850 m a.s.l.** | |
|  |  | **Wet to dry** | **Dry to wet** |  | **Wet to dry** | **Dry to wet** |  | **Wet to dry** | **Dry to wet** |  | **Wet to dry** | **Dry to wet** |
| **Trees** | **Abundance** | 802 | |  | 438 | |  | 511 | |  | 274 | |
|  | **Species richness** | 62 | |  | 32 | |  | 32 | |  | 16 | |
|  | **Sampling coverage** | 0.98 | |  | 0.99 | |  | 0.99 | |  | 0.99 | |
| **Butterflies** | **Abundance** | 74 | 67 |  | 355 | 95 |  | 255 | 193 |  | 68 | 119 |
|  | **Species richness** | 25 | 23 |  | 8 | 5 |  | 21 | 29 |  | 4 | 5 |
|  | **Sampling coverage** | 0.88 | 0.85 |  | 0.99 | 0.99 |  | 0.97 | 0.95 |  | 1.00 | 0.99 |
| **Fruit-feeding moths** | **Abundance** | 1,806 | 184 |  | 458 | 93 |  | 192 | 499 |  | 101 | 144 |
|  | **Species richness** | 85 | 39 |  | 60 | 24 |  | 55 | 92 |  | 19 | 32 |
|  | **Sampling coverage** | 0.98 | 0.91 |  | 0.94 | 0.85 |  | 0.86 | 0.92 |  | 0.94 | 0.85 |
| **Light-attracted moths** | **Abundance** | 326 | 61 |  | 633 | 473 |  | 208 | 469 |  | 383 | 597 |
|  | **Species richness** | 30 | 19 |  | 45 | 52 |  | 62 | 101 |  | 40 | 38 |
|  | **Sampling coverage** | 0.97 | 0.84 |  | 0.98 | 0.96 |  | 0.86 | 0.90 |  | 0.96 | 0.99 |

**Table S2.** Summary of the redundancy analyzes (RDA) analysing the effect of interaction between *disturbance* by forest elephant and *elevation* for trees, butterflies, and light-attracted moths species richness of families with ≥5 species. For butterflies and light-attracted moths, the temporal variation was treated by adding *season* as a covariate. See biplots in Supplementary Fig. S1.

| **Statistic** |  | **Axis 1** | **Axis 2** |
| --- | --- | --- | --- |
| ***Trees*** |  |  |  |
|  | Adjusted explained variation: 52.3% |  |  |
|  | Eigenvalues | 0.4 | 0.2 |
|  | Explained variation (cumulative) | 36.3 | 56.0 |
|  | Pseudo-canonical correlation | 0.9 | 0.8 |
|  | Explained fitted variation (cumulative) | 64.0 | 98.4 |
|  |  |  |  |
| ***Butterflies*** | |  |  |
|  | Adjusted explained variation: 36.4% |  |  |
|  | Eigenvalues | 0.4 | 0.03 |
|  | Explained variation (cumulative) | 35.9 | 39.3 |
|  | Pseudo-canonical correlation | 0.9 | 0.5 |
|  | Explained fitted variation (cumulative) | 91.0 | 99.6 |
|  |  |  |  |
| ***Light-attracted moths*** | |  |  |
|  | Adjusted explained variation: 49.1% |  |  |
|  | Eigenvalues | 0.3 | 0.2 |
|  | Explained variation (cumulative) | 29.6 | 47.2 |
|  | Pseudo-canonical correlation | 0.9 | 0.8 |
|  | Explained fitted variation (cumulative) | 56.4 | 90.05 |


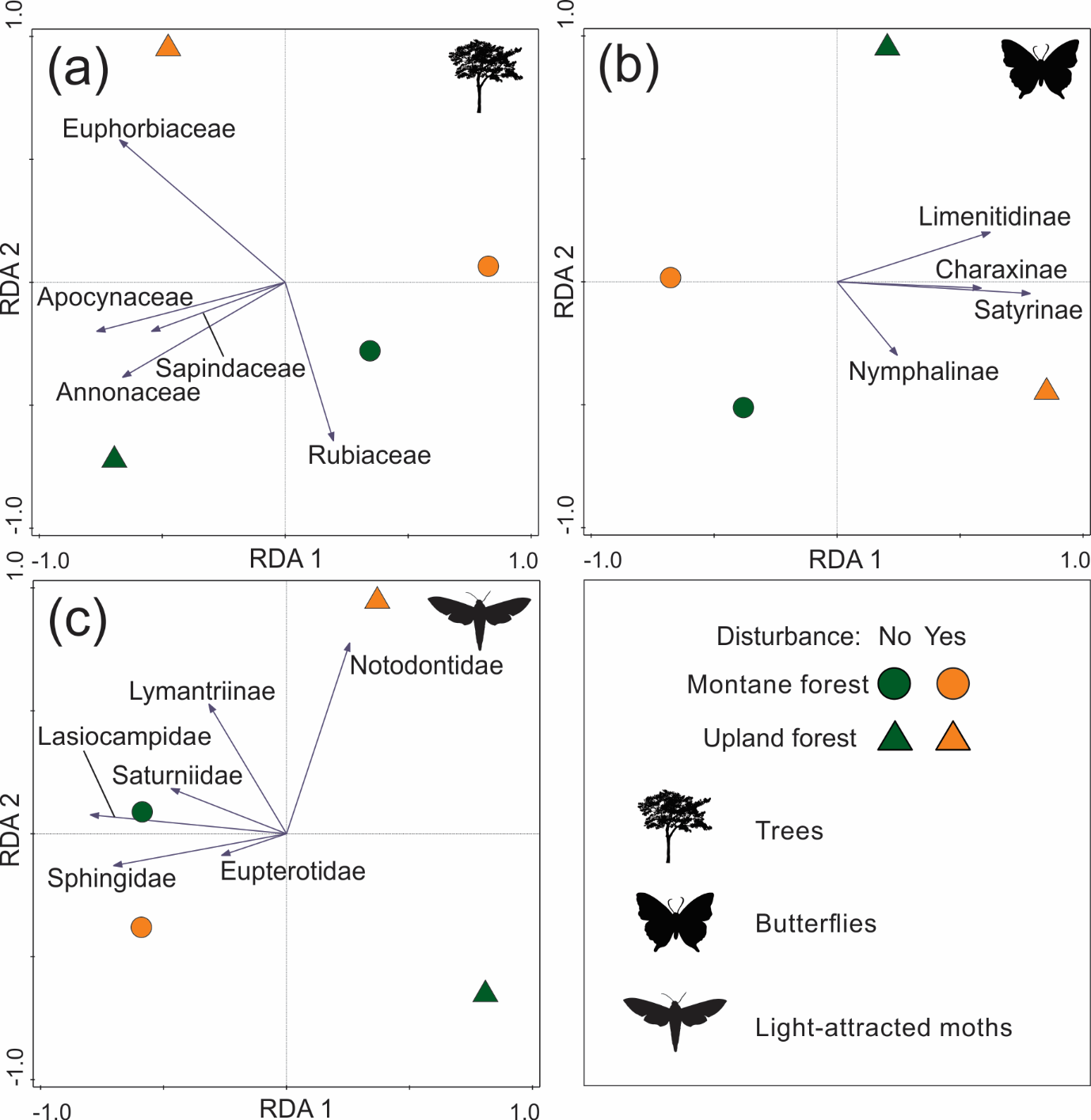


**Figure S1.** RDA ordination diagrams visualizing the effect of interaction between *disturbance* by elephants and *elevation* for species richness of families of (a) trees, (b) fruit-feeding butterflies, and (c) light-attracted moths. For butterflies and light-attracted moths, the temporal variation was treated by adding season as a covariate. See detailed results in Supplementary Table S2.
